# Overcoming big bottlenecks in vascular regeneration

**DOI:** 10.1038/s42003-024-06567-x

**Published:** 2024-07-18

**Authors:** Dalia A. Fantini, Guang Yang, Astha Khanna, Divya Subramanian, Julie A. Phillippi, Ngan F. Huang

**Affiliations:** 1 Department of Bioengineering, Swanson School of Engineering, University of Pittsburgh, Pittsburgh, PA USA; 2 Department of Cardiothoracic Surgery, Stanford University, Stanford, CA USA; 3 Epicrispr Biotechnologies, Inc, South San Francisco, CA USA; 4 Graver Technologies, Newark, NJ USA; 5 Department of Bioengineering, University of Texas at Dallas, Richardson, TX USA; 6 Department of Cardiothoracic Surgery, University of Pittsburgh School of Medicine, Pittsburgh, PA USA; 7 McGowan Institute for Regenerative Medicine, University of Pittsburgh, Pittsburgh, PA USA; 8 Stanford Cardiovascular Institute, Stanford, CA USA; 9 Department of Chemical Engineering, Stanford University, Stanford, CA USA; 10 Veterans Affairs Palo Alto Health Care System, Palo Alto, CA USA

**Keywords:** Tissues, Cardiac device therapy

## Abstract

Bioengineering and regenerative medicine strategies are promising for the treatment of vascular diseases. However, current limitations inhibit the ability of these approaches to be translated to clinical practice. Here we summarize some of the big bottlenecks that inhibit vascular regeneration in the disease applications of aortic aneurysms, stroke, and peripheral artery disease. We also describe the bottlenecks preventing three-dimensional bioprinting of vascular networks for tissue engineering applications. Finally, we describe emerging technologies and opportunities to overcome these challenges to advance vascular regeneration.

## Abstract (web summary)

Authors discuss big bottlenecks that inhibit vascular regeneration in the disease context of aortic aneurysm, stroke, and peripheral artery disease, along with emerging opportunities to overcome these challenges.

## Introduction

**Fig. 1:**
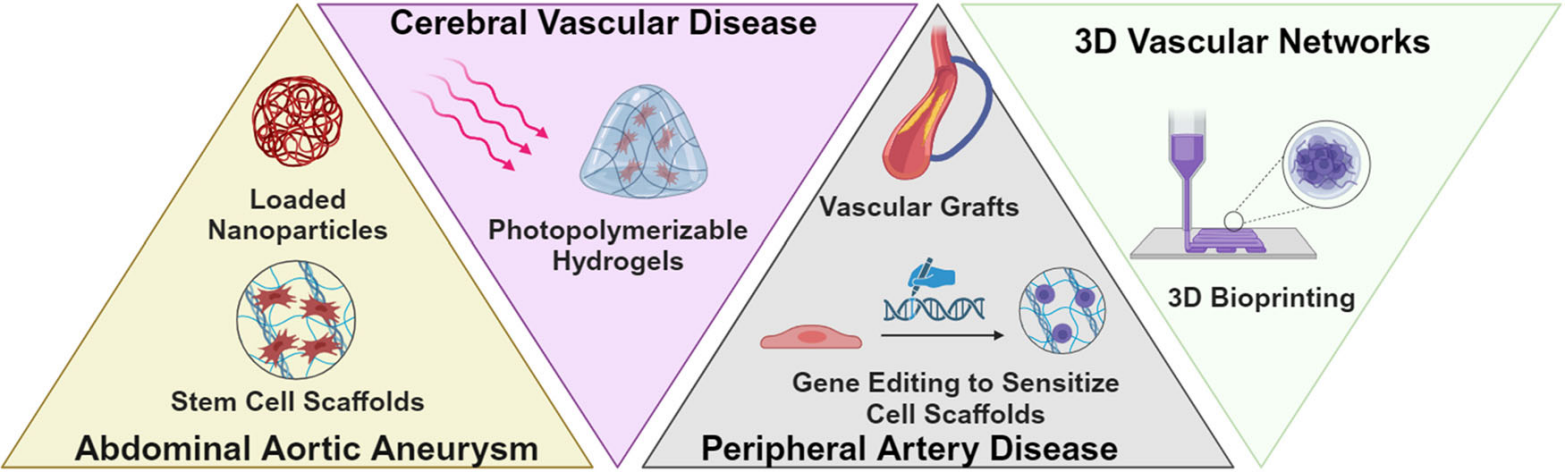
Overview of bioengineering and regenerative medicine approaches to promote vascular regeneration. Created with BioRender.com.

Cardiovascular diseases (CVDs) account for nearly 18 million deaths worldwide, according to the World Health Organization^1^. Among them include diseases that impair the vascular system such as aortic aneurysm, peripheral artery disease (PAD), and stroke. Vascular diseases commonly arise from the dysfunction of small or large blood vessels. Capillaries, the smallest form of vessels, comprise a monolayer of endothelium. In larger vessels such as arteries and veins, the endothelium is further surrounded by layers of medial smooth muscle and an outer layer of adventitia comprised of heterogeneous cell populations and a microvascular network known as the vasa vasorum or “vessels of vessels.” Although the pathology of vascular disease varies, they all share the common factor in which vascular regeneration is a promising therapeutic strategy to combat these diseases. Vascular regeneration approaches can be advanced using broad technologies including those from the fields of bioengineering, biomaterials, and regenerative medicine. Here, we comment on big bottlenecks that inhibit vascular regeneration in aortic aneurysms, peripheral arterial disease (PAD), and stroke by discussing the gaps in knowledge that prevent the advancement in new therapies (Fig. 1). Additionally, since three-dimensional (3D) bioprinting of vascular networks can benefit vascular regeneration in a variety of disease contexts and ameliorate tissue ischemia, we further describe the bottlenecks limiting clinical translation of 3D bioprinting for engineering vascularized tissues (Box. 1).

Box 1 Current limitations and potential bioengineering solutions

**Table:** 

Disease	Current Limitations	Potential Bioengineering Solutions
Aortic aneurysm	Chronic inflammation and elastin degradation	Penta galloyl glucose (PGG)-loaded nanoparticles stabilize elastin and resist MMP degradation Stem cell therapy secretes paracrine factors that improve aneurysmal remodeling Engineered biomaterials to provide structural support or as carriers for cell delivery Engineered vascular grafts that possess elastin organization
Ischemic Stroke	Impaired vascular regeneration	Extracellular nanovesicles induce blood vessel density, macrophage polarization, and attenuation of neuronal damage Biomimetic nanovesicles can further target specific signaling pathways and cell interactions Biomaterials improve cell infiltration and a reduce lesion volume
Peripheral artery disease	chronic inflammation and impaired vascular perfusion	Immunomodulatory vascular grafts that regulate inflammation through engineered cells or signaling factors Biomaterials with immunomodulatory mechanical properties Engineered angiogenic stem cells with anti-inflammatory factor overexpression

## How can we initiate elastin regeneration in an abdominal aortic aneurysm (AAA)?

Aortic aneurysms, which are characterized by an expansion of the aorta resulting from tissue degeneration, affect 5-10 individuals for every 100,000 in the US each year and carry a high rate of mortality^2^. The enlargement of the aortic wall that is characteristic of aneurysm progression is associated with the degeneration of the tunica media, loss of arterial wall function, decrease in extracellular matrix (ECM) integrity and the loss of native functional cells^3–5^. The close association of the structural proteins like elastin and collagen, along with smooth muscle cells, gives rise to the viscoelastic properties and static and dynamic properties of the aortic wall^3–5^. The inciting processes of medial tissue degeneration during aortic aneurysm remains unknown. However, aortic aneurysm development and progression are suspected to be the result of chronic inflammation, with the infiltration of neutrophils and macrophages within the aortic aneurysm site^6^. The current treatment methods for aortic aneurysm do not focus on mitigating the immune response or regenerating arterial tissue, but instead aim to physically reinforce the aortic wall by graft placement to restore the biomechanical properties of the tissue. Despite early promise from work in murine models of aortic aneurysm, pharmacological approaches have no demonstrated effect on aortic aneurysm regression in human clinical trials^7,8^. Most aortic aneurysm investigations and construction of in vivo models have been developed for abdominal aortic aneurysm (AAA), with a lesser emphasis on thoracic aortic aneurysms.

One bioengineering strategy involving penta galloyl glucose (PGG)-loaded nanoparticles for the treatment of AAA has been explored extensively and is being developed commercially as a potential treatment^9–12^. Early in vitro studies found that PGG nanoparticles are resistant to macrophage^13^ and vascular smooth muscle uptake^14^, with no significant impact on the viability of rat aortic endothelial and smooth muscle cells^13^. Initial pre-clinical studies involved the local periadventitial delivery of non-cytotoxic amounts of PGG during elastase and CaCl_2_-induced aneurysm development in mice^9,10^. PGG was demonstrated to strongly bind to strictly elastin fibers, thus stabilizing elastin and prohibiting further development of aneurysm, even in the presence of matrix metalloproteases (MMPs) secreted by inflammatory cells^9,10^. The commercialized PGG system known as the Nectero Endovascular Aneurysm Stabilization Treatment (EAST)® system will soon undergo human clinical trials as a new therapeutic strategy to treat AAA. Protection of endogenous aortic elastin by PGG allows for stabilization of local tissue remodeling and improvements to vascular function. The primary study endpoints will be the successful insertion and delivery of PGG in the absence of major events after one month^15^. Despite a mix of skepticism and enthusiasm for this therapeutic strategy, the results of this clinical trial should reveal new insights and inform future strategies.

An additional extensively explored therapeutic strategy involves the delivery of stem cells, such as adipose-derived and bone marrow-derived mesenchymal stromal cells, resulting in regression of the aneurysmal disease state in part by paracrine mechanisms^16–20^. The delivery modalities of these stem cell therapies include peri-adventitial and intravenous approaches, although peri-adventitial delivery of cells is more favorable due to cell migration and engraftment^16–20^. Peri-adventitial delivery of human induced pluripotent stem cell (iPSC)-derived smooth muscle progenitors and primary smooth muscle cells via a collagen scaffold^21^have been examined in a murine model of AAA for the promotion of vascularization. Implementation of a bioengineered or commercially available scaffold construct promotes the localization of cells to the site of the aneurysm and provides ECM cues that foster transplant cell survival. Despite early enthusiasm on the potential of cell therapy, the peri-adventitial cell delivery modality requires invasive surgical procedures like a laparotomy, which involves a large surgical incision to access the abdominal cavity. Alternative strategies that involve systemic delivery of cells may also have translational relevance^20^, although numerous other obstacles persist including the mode of delivery, tissue-homing capacity, transplant cell viability, and scaling/manufacturing and regulatory hurdles. Together, these studies suggest that the transplantation of therapeutic cells, in conjunction with biomaterials, may be a promising strategy to abrogate the progressive dilatation associated with aortic aneurysms.

Another strategy involves vascular grafts and tissue constructs for endovascular repair of abdominal aneurysm (EVAR) and Bentall procedures. However, these grafts typically require invasive surgical procedures. Furthermore, these conduits do not protect existing elastin or encourage endogenous elastin production, nor do they modulate an immune response. Tissue-engineered vascular grafts with regenerative properties developed by Humacyte demonstrate the feasibility for treating vascular repair. The Human Acellular Vessel (HAV) is composed primarily of human collagen and other ECM components and has been examined in Phase II and III clinical trials for hemodialysis and vascular trauma^22^ but not yet for treatment of AAA. It is possible that bioactive vascular grafts can be similarly developed for AAA mitigation, while retaining the necessary compliance and other biomechanical properties required for aortic function.

Additionally, the development of preventative personalized medicine solutions specifically for aortic aneurysm is complicated by poor early detection. Screening of at-risk populations using ultrasound is understood to be the best method of aneurysm prevention. However, the lack of patient participation in these screenings is a predominant issue for physicians. Transgenic models and multi-omics are useful tools to map disease pathways for a comprehensive mechanistic understanding. These tools can be used specifically for understanding the cytokine signaling pathways and MMPs that contribute to elastin degradation and tissue deterioration. In summary, regenerative medicine strategies involving therapeutic cells and engineered vascular grafts, along with advanced multi-omics strategies and personalized medicine, lead the way towards overcoming bottlenecks in the treatment of aortic aneurysms.

## How can tissue engineering approaches revert cerebral dysfunction due to stroke?

Affecting 795,000 people in the US each year, a stroke is regarded as the inhibition of oxygen delivery to brain tissue, resulting in neuronal cell apoptosis or necroptosis due to the presence of reactive oxygen species^23–25^. Impaired blood flow may be attributed to vessel blockage due to an embolus or thrombus, resulting in ischemic stroke or a vessel rupture and subsequently a hemorrhagic stroke^24,25^. Current treatment methodologies for ischemic stroke include systemic or catheter-directed thrombolysis, which is dependent on drug efficacy and the size and location of the clot^24^. In the instance of a ruptured aneurysm, surgical intervention may be utilized to initiate clotting at the aneurysm site or redirect the flow of blood away from the ruptured vessel^25^. Although these treatment methods may mitigate additional brain tissue damage and allow for the restoration of blood flow, there is a clear clinical need for the recovery of damaged tissue, which may compromise a patient’s ability for motor and cognitive rehabilitation^26^.

Current bioengineering technologies for ischemic stroke focus on the neuro-regeneration of brain tissue located proximal to the stroke site by cell-, nanoparticle-, and biomaterials-based approaches. Extracellular nanovesicles derived from mesenchymal stem cells with magnetic properties have been explored for targeting ischemic lesions to promote angiogenesis, anti-apoptosis, and anti-informatory responses by upregulating the expression of select therapeutic molecules^27^. This approach has proven successful in a middle cerebral arterial occlusion (MCAO) ischemia-reperfusion murine model with demonstrated localization of nanovesicles to the ischemic brain lesion, resulting in upregulation in blood vessel density, macrophage polarization, and attenuation of neuronal damage^27^. The delivery of nitric oxide-boosted and activated nanovesicles demonstrated the ability for nitric oxide storage in endothelial cells to recruit pericytes upon nitric oxide release^28^. Facilitation of pericyte and endothelial interactions allows for the revascularization at the site of injury. It was shown that biomimetic nanovesicles composed of red blood cells and platelet plasma membrane containing hypoxia inducible factor in a MCAO model preserved the integrity of the blood brain barrier and promoted angiogenesis by improving microcirculation^29^. The localization of therapeutics to the disease site remains a concern for clinical implementation. However, the implementation of biomimetic or magnetic nanovesicles may be extended to other disease conditions that may not be readily operable.

The use of urinary bladder-^30^ and brain-derived ECM^31^ has been explored for traumatic brain injuries and stroke for the treatment of tissue loss. ECMs (both gelling and non-gelling) support endogenous cell infiltration and a reduction in lesion volume^32^, regardless of the type of ECM used (endogenous to the region or otherwise). Preliminary in vitro and in vivo work reveals promise for these regenerative medicine technologies for the targeting and reversion of afflicted brain tissue post-ischemic inflammation by addressing tissue loss and local immune and endogenous cell responses.

Current medical intervention for ischemic stroke includes immediate surgical intervention and medications to prevent clot formation. However, the approaches discussed above offer modes of regenerating brain tissue and aiding in the repair of the blood brain barrier. Future directions include combinatorial therapies, which may vary from patient-to-patient and would be dependent on the severity and cause of the stroke. Developments in screening and mapping disease pathways through artificial intelligence and multi-omics would give physicians the tools to treat patients using these minimally invasive methods. Artificial intelligence has been previously explored for the processing of medical images relating to ischemic stroke diagnostics and screening. Examples include fluid attenuated recovery inversion recovery magnetic resonance imaging^33^ and computed tomography angiography^34^, citing improvements in accuracy and timeliness of results^33,34^. Further developments in artificial intelligence for diagnostics and medical imaging analysis would be significant for other vascular diseases, such as aortic aneurysm, which has recently been explored^35^.

## How can we manipulate inflammation at the sites of peripheral artery disease to accelerate angiogenesis?

In the US alone, the prevalence of peripheral artery disease (PAD) is estimated to be more than 8.5 million adults, accounting for 12–20% of individuals over the age of 60 years^36^. PAD is accountable for high medical expenditure and mortality^37^. PAD is associated with reduced blood perfusion to the limbs that can result in limb threatening tissue ischemia, in severe cases requiring limb amputation. Inflammation is crucial in both the onset and progression of atherosclerosis that causes PAD pathogenesis^38^. Several pro-inflammatory cytokines are found strongly associated with PAD, such as plasma interleukin-6 (IL-6), tumor necrosis factor-α (TNF-α), and monocyte chemoattractant protein-1^39^. In addition, vascular endothelial cells may be activated for inflammatory functions^40^. In response to inflammation, E-, L-, and P-selectins are significantly elevated in PAD patients. Likewise, MMP-2 and MMP-9, known as the major gelatinases interacting with arterial matrices in oxidative stress situations, are highly enriched in plasma drawn from patients with PAD^41–44^.

Evidence suggests that inflammation and hypoxia are closely related with angiogenesis, which otherwise rarely occurs spontaneously in adults^45^. Upon exposure to pro-inflammatory cytokines, vascular endothelial cells detach from each other and then destabilize from the underlying basement membrane. This process is mediated by MMPs and enables cell migration via integrin-mediated adhesion to ECM proteins. The migrating and proliferating endothelial cells constitute cord-like structures in target tissues, which will later create lumens to form functional vessels jointly with surrounding ECM and supportive cells^46^.

Tissue engineered vascular grafts have garnered much research interest in the past decades to facilitate timely arterial occlusion bypass or blood flow restoration for PAD patients^47,48^. Traditional strategies focused on engineering vascularized grafts through pre-seeding of endothelial cells into the luminal layer to ameliorate thrombosis^49^. Given the multifaceted association between inflammation and angiogenesis, recent directions also consider the role of an inflammatory milieu on the therapeutic response of cell-seeded scaffolds. In particular, the ability of vascular grafts to respond to the host inflammatory environment may be beneficial for accelerated functionalization and vascularization while avoiding any function loss due to inflammatory stimuli.

Transient activation of interleukin (IL)-11 or IL-21 expression from engineered endothelial cells is likely to potentiate angiogenesis in PAD in a STAT3-dependent fashion^50,51^. Likewise, overexpressing vascular endothelial growth factor receptor-2 (VEGFR2) in cells seeded on scaffolds may augment vascular endothelial growth factor (VEGF) signaling to accelerate hypoxia-mediated angiogenesis and downstream signaling^52,53^. Moreover, multiplexing or orthogonal gene editing of the Angiopoietin-Tie2 axis may orchestrate the survival and activation of pre-seeded endothelium on scaffolds that would otherwise remain quiescent^54^ in response to tissue ischemia to ensure maximal blood vessel regeneration.

Biological scaffolds can provide physical support for cell attachment to build an inflammation-responsive vascular graft. With proper engineering, scaffolds harbor great potential to overcome the limitations of therapeutic cells exposed to an inflammatory PAD. For example, the vascular graft may be pre-loaded with an antagonist or silencer of IL-6 receptor to alleviate a potential hyperreactive response to the excessive IL-6 production^55^ that may cause defective angiogenesis^56^, compared to that stimulated by VEGF^57^. In addition, the topographical and chemical properties of the scaffold are involved in tuning the cellular response to the inflammatory environment. For example, the surface braiding patterns and poly(glycerol sebacate) coating on scaffolds have been found to influence MMP activity and tissue regrowth when implanted as an aortic graft^58^. Similarly, collagen scaffolds modified by chondroitin sulfate was shown at the implant site to augment the deposition of fibronectin, which confers a pro-regenerative environment^59^.

Strategies that concurrently modulate angiogenesis and inflammatory response are promising for the treatment of PAD. For example, 3D gelatin hydrogels with tunable micro- or macro-scale channel networks were shown to differentially induce microvascular formation and inflammation in a murine hindlimb ischemia model^60^. In particular, micro-channel hydrogels preferentially induced greater microvascular network formation and M2 macrophage polarization, compared to macro-channel hydrogels. Genetic modification of angiogenic stem cells is another approach to impart immunomodulation. For example, murine adipose-derived stem cells (ADSCs) that overexpress C-X-C motif chemokine receptor-4 (CXCR4) enhanced angiogenesis in a murine limb ischemia model^61^. This finding was supported by gene expression analysis of CXCR4-overexpressing ADSCs cultured in hypoxia in vitro that demonstrated an upregulation of angiogenic genes encoding vascular endothelial growth factor and hepatocyte growth factor, along with a downregulation of pro-inflammatory markers IL-1 and IL-6. These studies highlight the therapeutic benefit of cell and/or biomaterial treatments that regulate both angiogenesis and inflammation in the setting of tissue ischemia.

The emergence of new biotechnologies may drive breakthroughs in the future. For example, cell fate trajectory analyses of single cell sequencing data may reveal previously unknown master genes that dictate angiogenic behaviors of cells in an inflammation milieu, thereby potentially identifying novel targets for gene editing. The development of advanced gene editing tools such as Clustered Regularly Interspaced Short Palindromic Repeats (CRISPR) epigenetic editors may advance the engineering of biological vascular grafts in a multiplex or even orthogonal fashion to coordinate inflammation and angiogenesis in PAD. Finally, machine learning-based analysis of circulating proteomic panel from PAD patients may classify distinct inflammation phenotypes to inform therapy selection for more precise, personalized vascular graft preparation and treatment. We envision that these technological breakthroughs will deepen our understanding of the inflammatory milieu in PAD and facilitate the corresponding engineering of vascular graft implantation.

## What obstacles limit the creation of vascular networks using 3D bioprinting?

Despite advancements in tissue engineering and regenerative medicine for restoring diseased or dysfunctional tissues, the engineering of a functional and mature microvascular network in 3D tissues to allow for adequate perfusion of oxygen and growth factors remains an unmet need. Angiogenesis and the generation of a functional vascular bed have been studied by spatiotemporal approaches of vascularization through the microfabrication of complex vascular networks, the coordinated orchestration of angiogenic factors, and the differentiation of vascular cells^62^. 3D bioprinting is a multidisciplinary technique that utilizes bio-inks to spatially pattern biological and cellular constituents to create 3D organ analogs and tissues^63^. 3D bioprinting has diverse applications in regenerative medicine, drug screening, and personalized medicine. Technological advancements in FRESH (Freeform Reversible Embedding of Suspended Hydrogels) bioprinting now enable the bioprinting of a human heart model or perfusable vasculature, although not at the level of physiological complexity^64–66^.

Despite the recent advancements in 3D bioprinting that have made tremendous progress in generating more biomimetic vascular networks, there remains challenges that need to be addressed for successful clinical translation of engineered vascularized tissues using 3D bioprinting technology. The first challenge is the ability to generate dense capillary networks for efficient oxygen diffusion and nutrient transport, as passive diffusion is usually limited to 200–250 µm in tissues^67^. This restricts the thickness of constructs that rely primarily on passive diffusion. To overcome this issue, sophisticated microfluidic systems and microscale technologies can be incorporated for in vitro generation of microvascular networks. Another challenge lies in the non-uniform and non-homogeneous cellular infiltration within bioprinted constructs. However, strategies that enable therapeutic cells to be bioprinted within porous biomaterials might ameliorate diffusion concerns within the 3D structure.

A technical limitation of 3D bioprinting is the ability to overcome directional flow conditions of physiological shear stress^68^. During the bioprinting process, shear stress applied to cells can impair cellular growth, thereby adversely altering gene expression and cellular function^68^. Vascular endothelial cells are sensitive to such mechanical forces and result in diminished cell viability. Cells encapsulated within shear-thinning hydrogels at the time of bioprinting can be protected from the shear forces generated during printing that also maintain their functional activity^69^ An additional challenge is vascular anastomosis, in which the bioprinted vascular network integrates with the host circulation for effective blood perfusion after implantation. Although the biological signaling cues that promote anastomosis has been studied, the ability to induce sufficient anastomoses has been difficult to achieve in practice^70^.

We anticipate that technological developments are needed to improve the spatial resolution and complexity of bioprinted tissues. A notable thrust area is laser patterning bioprinting with light sheet imaging that incorporates live imaging of the bioprinted tissue with high resolution and high-speed capabilities. This 3D bioprinting strategy utilizes a light sheet-based system to photo-crosslink polymers into hydrogels at a high speed and resolution (~9 µm)^71^. This bioprinter can track cells and bio-ink during crosslinking for real time evaluation of 3D bioprinted constructs. As a light-based bioprinter, this technology is promising for generating customized 3D bioprinted structures with long-term cell culture viability. Another technology with translational potential is multi-photon printing^72^, which can be applied to generate spatially complex biomaterials sub-micron features. Multi-photon technique can be further complexed with intravital bioprinting, thereby enabling real-time bioprinting of physiologically relevant geometries with precision placement in vivo^73^. With these technological developments, we anticipate that 3D bioprinting of de novo organs will one day reach clinical translation.

## Forward-Looking Outlook

In conclusion, bioengineering and regenerative medicine strategies hold great potential in advancing vascular regeneration across a range of vascular diseases, including aortic aneurysm, stroke, and PAD. However, next generation regenerative therapies should be developed while keeping in mind the scalability of tissue engineered constructs, the post-implantation viability and immune response, and the regulatory process towards clinical testing. In conjunction with other state-of-the-art technologies such as gene editing and the integration of artificial intelligence for personalized medicine and multi-omics analysis, these bioengineering strategies may one day overcome the current bottlenecks in vascular regeneration.

### Reporting summary

Further information on research design is available in the Nature Portfolio Reporting Summary linked to this article.

### Supplementary information

Reporting Summary
